# Exploring implicit bias in the perceived consequences of prematurity amongst health care providers in North Queensland – a constructivist grounded theory study

**DOI:** 10.1186/s12884-021-03539-5

**Published:** 2021-01-13

**Authors:** Susan Ireland, Robin Ray, Sarah Larkins, Lynn Woodward

**Affiliations:** 1 James Cook University, College of Medicine and Dentistry, Angus Smith Drive, Townsville, Australia; 2 Neonatal unit, Townsville University Hospital, Townsville, Australia

**Keywords:** Pregnancy, Extreme prematurity, Resuscitation, Counselling, Attitudes, Implicit bias

## Abstract

**Background:**

A study was done to explore the attitudes of relevant health care professionals (HCP) towards the provision of intensive care for periviable and extremely premature babies.

**Methods/design:**

Applying a constructivist grounded theory methodology, HCP were interviewed about their attitudes towards the provision of resuscitation and intensive care for extremely premature babies. These babies are at increased risk of death and neurodisability when compared to babies of older gestations. Participants included HCP of varying disciplines at a large tertiary centre, a regional centre and a remote centre. Staff with a wide range of experience were interviewed.

**Results:**

Six categories of i) who decides, ii) culture and context of families, iii) the life ahead, iv) to treat a bit or not at all, v) following guidelines and vi) information sharing, emerged. Role specific implicit bias was found as a theoretical construct, which depended on the period for which care was provided relative to the delivery of the baby. This implicit bias is an underlying cause for the negativity seen towards extreme prematurity and is presented in this paper. HCP caring for women prior to delivery have a bias towards healthy term babies that involves overestimation of the risks of extreme prematurity, while neonatal staff were biased towards suffering in the neonatal period and paediatricians recognise positivity of outcomes regardless of neurological status of the child. The implicit bias found may explain negativity towards intensive care of periviable neonates.

**Conclusion:**

Understanding the presence and origins of role specific implicit bias may enable HCP to work together to improve care for parents preparing for the delivery of extremely premature babies.

**Supplementary Information:**

The online version contains supplementary material available at 10.1186/s12884-021-03539-5.

## Background

The gestation at which a baby can survive has reduced with the evolution of increasingly complex intensive care [1]. At the lowest gestations, there is a higher risk of death and poor neurological outcomes in those who survive compared to those of longer gestation [2–5]. These babies are often referred to as periviable [1, 6, 7]. Periviable babies may receive either palliative care with comfort measures only following delivery, or be offered full intensive care. The care a baby receives will depend on collaborative decision-making by the parents and health care professionals, after consideration of individual factors pertaining to the pregnancy and baby [8]. There is some variation in the gestations used to determine when it is considered appropriate to offer resuscitation depending on the country of birth and local organisational recommendations. In Australia, many guidelines deem intensive care to be inappropriate below 23 weeks completed gestation, a ‘grey area’ exists from 23 to 24 + 6 weeks where resuscitation may be considered. From 25 weeks completed gestation, most guidelines used in high income countries suggest that resuscitation should usually occur unless there are specific adverse factors which would increase the risks of a poor outcome. Factors that are considered include expected birth weight, gender, plurality, chorio-amnionitis and congenital abnormalities [9–11]. Increasingly, other countries are recognising improved outcomes at 22 weeks gestation where active care is offered [5, 12, 13].

Few parents who find themselves in the position of having to participate in decision making at extremely preterm gestations have the medical knowledge required to make these decisions without the counselling of health care practitioners (HCP) [14, 15]. Parents are often still coming to terms with the situation and rely on both information and counselling from the HCP which includes an exploration of parental experience and beliefs [6, 16]. Traditionally, studies have regarded obstetricians and neonatologists as the main sources of information for parents. However, it is apparent that midwives, neonatal nurses and allied health staff also provide support and interpretation for parents [17, 18]. Studies have shown that most HCP are inaccurate in their perceptions of the rates of survival and intact survival for those babies in the lowest gestations [19–22]. The reasons for this inaccuracy are poorly understood. Although HCP bias with regards to active care according to gestational age has frequently been explored in terms of the different groups of HCPs who offer support to the parents [23, 24], the origins of this bias are less well documented.

Implicit bias is a subconscious attitude formed by the persons’ own background and life experiences, which negatively influences behaviour [25, 26]. Implicit bias is well recognised in medical literature where the effects on racial and social disparities has been the focus of research [27, 28]. A meta-analysis of studies shows a positive correlation between implicit bias and lower quality of care [29].

This paper describes the exposure of role specific implicit bias amongst HCP and the possible contributing factors for this bias. These findings are a component of a larger study investigating attitudes towards extreme prematurity.

### Context for the study

Townsville University Hospital contains the only neonatal intensive care unit (NICU) in North Queensland and provides care for all neonates under 28 weeks completed gestation for an area of 500,000 km^2^. Ten thousand babies are born in the region annually. Approximately 2500 to 3000 deliveries occur per year at TUH, with 40–50 extremely preterm babies admitted each year. Over 50% of the babies admitted for extreme prematurity will live outside the immediate tertiary centre catchment area, with 25% retrieved following birth at other centres [30]. Two to four extremely premature babies are born at the regional and remote centres studied per year. Retrieval of outborn babies is performed by a dedicated retrieval service based at the NICU. The regional maternal-foetal medicine (MFM) unit and paediatric surgical services are based in the tertiary centre. Aboriginal and Torres Strait Islander (hereafter referred to as Indigenous) people comprise just over 10 % of the North Queensland population, but constitute 38% of deliveries at extreme prematurity and 43% of retrievals [30]. There are higher rates of poverty, remote residence and poor health outcomes for the Indigenous population in North Queensland [31] than for the non-Indigenous population.

Current Queensland guidelines indicate that resuscitation below 23 weeks should be discouraged, and babies over 25 weeks gestation should receive intensive care unless there are known congenital anomalies [9]. From 23 completed weeks to 23^+ 6^ weeks parents should be the final arbiters of decisions to offer active care, while a ‘fully informed’ parent may choose to decline active care from 24 to 24^+ 6^ gestation. Women at risk of delivering babies early will initially have contact with the midwives and obstetricians as complications of the pregnancy develop. They receive counselling from these staff who will then refer the woman and her partner to the neonatal staff for further counselling about the outlook for the baby. Obstetric and neonatal staff then work with the family to establish a plan for the delivery and care of the extremely preterm infant. Where possible, the families visit the NICU prior to delivery, with neonatal nurses providing the tour and later the nursing care for the baby following admission. Social workers and Indigenous Liaison Officers provide support for families and are often present for discussions between HCP and families. Following resuscitation, the baby is transferred to the neonatal intensive care unit, but if there are complcations which increase the risks of long term neurodisability, care can be redirected from intensive care to palliation and the baby will die. This is considered legal and ethical in this jurisdiction. At antenatal counselling this option is frequently offered. After discharge from the neonatal unit, all extremely preterm babies will be cared for by their local paediatric services.

Referral centres to the tertiary unit include two large regional hospitals 350 km to the north and south which can provide care for babies from 29 weeks and 32 weeks respectively. There is a small remote hospital located 900 km away, near the western border of the state. Full time obstetric and paediatric staff are in the three main referral centres. Other birthing facilities staffed by general practitioners with obstetric qualifications and midwives are scattered around the North Queensland area. Rarely a baby will be born at a health centre staffed only by experienced rural nurses. Antenatal transfer to the tertiary unit for women at risk of extreme preterm delivery occurs where possible.

## Methods

A convergent mixed methods study was undertaken to explore the attitudes of HCP towards the provision of intensive care for periviable and extremely premature babies in North Queensland. The quantitative component of this study has been published [32]. This qualitative study was informed by constructivist grounded theory methodology as described by Charmaz [33]. This methodology was chosen as the researcher first explores the behaviour or attitude which is studied and then builds theories about the underlying causes for these. Building theories to explain findings is useful in healthcare as it can lead researchers to suggest ways to change negative behaviours or enhance positive ones. Healthcare providers caring for pregnant women at risk of delivering extremely premature babies, or who care for the babies after birth, were interviewed to understand their attitudes towards the care for extremely preterm babies. The interviews followed an interview guide adapted through an iterative process of initial coding and focused theoretical interactions with the data to further explore tentative categories (see supplementary file 1). Interviews were analysed soon after they were done and the guide altered to ensure that the categories of findings was fully explored. The consolidated criteria for reporting qualitative studies (COREQ) [34] checklist item guide was followed.

### Sampling strategy

A pragmatic, purposive strategy was used to enrol participants from a tertiary, regional and remote hospital. A quantitative survey was sent to HCP who provide care for women at risk of extremely preterm delivery, and those caring for the babies after birth, to investigate their knowledge and attitudes towards the active care of extremely preterm babies. The survey commenced shortly before this qualitative study [32] and the two then ran contemporaneously with the qualitative study continuing for several months after the survey study was completed. An invitation to participate in the qualitative study was included in the quantitative study. One hundred and seventy-four invitations were sent for the survey, with 113 participants (64.9% participation rate). Separately, all full time obstetric, neonatal and paediatric specialist medical staff at the tertiary unit were invited individually by email to participate in the interviews. All invited HCP agreed to participate, although only three out of five obstetric and two of six paediatric staff participated as data saturation was reached once external participants in these groups of workers had been included. All three neonatologist participated. Outside the tertiary centre potential participants were identified by a local investigator and approached to ensure regional and remote representation in the study including three paediatricians, an advanced obstetric trainee and a neonatal nurse practitioner. The two remaining volunteers recruited by the regional and remote centre were not interviewed because data saturation was complete. The demographics of the participants were monitored contemporaneously. Further potential participants were then chosen (theoretically sampled) from the survey volunteers to ensure a range of HCP representing the demographics of experience, locality and health care roles, and to add to the emerging categorical data occurred. Six survey volunteers were not interviewed as they were not required. Age and ethnicity of participants were not recorded. In addition, a focus group was held involving two Indigenous Liaison Officers and an obstetric social worker, who together requested a focus group format rather than individual interviews. Recruitment ceased when ongoing analysis of the interviews as they occurred identified that no new data were emerging (data saturation) suggesting that theoretical adequacy and the emerging categories were complete.

### Data collection

Interviews were performed by the primary investigator (a neonatologist working at the tertiary centre who has training, experience and publications using qualitative research methodology) and a research assistant (a midwife researcher experienced in qualitative interviewing and who has published qualitative findings previously, but was not involved with the NICU). Immediate co-workers of the primary investigator (neonatal medical staff) were all interviewed by the research assistant, whilst all other participants were given the choice of interviewers. Interviews were conducted in the workplace or by telephone and recorded digitally.

The interviews explored the participants’ work experience and their experience in counselling patients at risk of extremely premature delivery. Opinions were sought about decision making around resuscitation of extremely premature babies both as a process and in terms of the actual factors the HCP would assess when offering intensive care. The relative roles of parents and HCP in decision making at specific gestations were explored. Participants were asked to offer any suggestions for improving decision making processes within the unit and offer any other comments which they might have about the care of periviable babies. Very early modification to the semi-structured interviews added questions specific to the Queensland Health guidelines and possible religious inclinations informing participants’ opinions.

Recorded interviews were transcribed by a commercial transcription service and returned to the research team within three days of the interview.

### Data analysis

Using NVivo as a data management software, interviews were analysed applying initial and focused coding enabling broad tentative categories to emerge. Focused codes were identified from the codes within the categories using a staged constant comparative process from focused coding to category generation. While the primary investigator did the initial coding, regular meetings were held where the study team examined the interviews and codes and discussed these to develop categories and build theories about the meaning of the findings. In this way analytic triangulation in the study team occurred and findings were also triangulated against the results of the quantitative study. Where disagreements were found in the interpretation, these were discussed until consensus was reached.

### Ethical approval

Ethical approval for the study was obtained from the Townsville Hospital and Health Service Human Research Ethics Committee and James Cook University (HREC/15/QTHS/194, JCU 6485). All participants received a written information letter about the research and consented to participate, recorded interviews and future publications.

## Results

Thirty-three HCP participated in the study (Table 1). Interviews lasted from 17 to 90 min.

**Table 1:** Demographic characteristics of participants

HCP role	Midwife	4 (12%)
	Neonatal nurse	5 (15%)
	Neonatal nurse practitioner	4 (12%)
	Obstetrician	3 (9%)
	Obstetric trainee	2 (6%)
	Neonatologist	3 (9%)
	Neonatal trainee	2 (6%)
	Paediatrician	5 (15%)
	Paediatric trainee	2 (6%)
	Allied health	3 (9%)
Experience in years	1–5 years	6 (18%)
	> 5–10 years	9 (27%)
	> 10–15 years	9 (27%)
	> 15–20	5 (15%)
	> 20	4 (12%)
Gender	Female	26 (79%)
	Male	7 (21%)
Location	Tertiary hospital	28 (85%)
	Regional and remote	5 (15%)
Interviewer	Primary investigator	22 (67%)
	Research assistant	11 (33%)

Categories which emerged included i) who decides, ii) culture and context of families, iii) the life ahead, iv) to treat a bit or not at all, v) guidelines and vi) information sharing. Whilst implicit bias based on racial and socioeconomic status was found within several categories, the concept of implicit bias towards prematurity itself emerged as a separate theoretical construct that is a theory to explain some of the findings. This manuscript will present the theory of implicit bias towards extreme prematurity with the contributing focussed codes: i) disability is a burden, ii) parents need protecting, iii) is the suffering just iv) uncertainty of outcome, v) disability in remote sites, vi) differing discipline perspectives, vii) influence of personal experience, and viii) evolving implicit bias.

### Disability is a burden

Termination of pregnancies because of known abnormality occurs, and the HCP working in the antenatal wards report familiarity with caring for women having this procedure. This midwife described her own experience caring for a woman whose baby had a condition which was not compatible with life, showing surprise that the woman would not end the pregnancy. She went on to describe familiarity with termination for other abnormalities. Caring for patients terminating a pregnancy was difficult for her, but her role was to support, and not get too emotionally close to the patient:

*… one baby that was twenty some - no I forget how many weeks she was. She was preterm and the baby had anencephaly and not compatible with life and she refused to terminate… I’ve had plenty. Lots of terminations for abnormalities and things like that…it’s really hard when you know someone is terminating on the ward. It’s really hard not to bond with them and it’s really hard. You’ve just got to support them and remember why you are here. Remember your role. I think you get better at it with practice. When I was a grad I was awful at it. I would tear up with the women and be a mess. (Junior midwife, HCP 20).*

Several of the obstetricians stated that the disability brought about by prematurity is a burden to families. They perceived the burden may lead to the clinician making decisions about active care. This clinician connected the concept of adequate counselling to declining resuscitation before the gestation she herself would choose:

*I mean it’s not up to me but again we’ll have to counsel the parents the right thing to do. I have seen a few women who after being properly counselled, understanding their long-term sequelae, say no, up to 25 weeks. Usually it didn’t involve the neonatal team…I make the decision on the long-term morbidity that the baby is going to have and the burden on the parents...All they want is the baby to be resuscitated but they don’t have things like on looking at the long term how the baby is going to do and what the neurological sequelae they could have like cerebral palsy and things…I try to give them information that is not just survival…if it was me and I was at 24 weeks and if I have a baby who is offered resuscitation I would say no up until I get to 25 weeks (Senior obstetric trainee, HCP 14).*

Whilst discussing resuscitation of babies of 23 and 24 weeks gestation, this obstetrician reflects her concern about the future potential burden of disability:

*I’m always terrified for my women that they are going to end up with a severely disabled kid that’s alive and that stays alive and they’re stuck with for life (Obstetrician, HCP 18).*

In the opinion of this obstetrician, even a lower risk of disability may lead a parent to prefer palliation after birth rather than active care. He felt that even above the guideline cut off at 25 weeks completed gestation, parents should be able to opt for comfort care only:

*I have issues around 25 weeks being the cut off where we must resuscitate because some parents might not wish that…then to insist that they’re resuscitated would, I think, be the wrong thing to do (Obstetrician, HCP 17).*

A neonatal nurse expressed how a baby for whom she had provided care has disabilities which she perceives as troubling:

*I have seen babies down the track who we’ve offered withdrawal of care and the parents have refused and have been severely disabled and its quite disturbing to see…It makes you think are we doing the right thing for these families (Neonatal nurse, HCP 2).*

### Parents need protecting

HCP suggested that parents require hope for a positive future for the baby in order to negotiate neonatal intensive care psychologically intact themselves. This hope may form a barrier to parents absorbing a more negative message which the HCP may be wanting to convey:

*I do think they get told in no uncertain terms what the situation is and what might happen…but you have that hope don’t you? And that’s the trouble with parents (Obstetrician, HCP 18).*

Paediatricians describe how they manage the child within the context of the family until late adolescence. They discussed how families appear to cope. Even where a child has disabilities, most families appear to adapt:

*So they struggle but it’s not that they ever said they would have changed anything…they seem happy with their children (Tertiary paediatrician, HCP 16).*

Some participants suggested that families adapt to disability gradually which allows the family to cope with the challenges involved in the care for their child. However the clinician needs to honestly assess the childs’ abilities which might lead to increased distress:

*It’s a journey…that concept of a child growing into their disability…as the physician we know with the history what we are expecting to see, but even if we have said that information to the families, most families will hold onto the positives which is important for positive coping.* (The parent is) *Looking at day to day gains…I see over a 6 to 12 month period as the child fails to meet developmental milestones that the grief continues. Sometimes it is augmented when you start talking about that difference we talked about as a possibility is happening now. (Paediatrician, HCP 15).*

Another clinician reflected how she would choose to dwell on the positives. Her reality has been that most parents will opt for resuscitation and her role is to stabilise the baby prior to transfer to the tertiary centre:

*Most of the time when we go to talk to them we usually try to be more positive than negative – when we think of a baby we are already going to resuscitate (Paediatrician, HCP 29).*

Caution about assessing a families’ ability to cope with disability prior to the birth was verbalised by a paediatrician:

*I don’t think we have all the information about families and until the family has been through the situation, you just don’t know (Paediatrician, HCP 15).*

### Is the suffering just?

Nursing staff and junior medical staff reported distress associated with caring for the extremely preterm babies and their part in causing the suffering. These staff all spend significantly more time daily with the baby than more senior medical staff. Two neonatal nurses commented below:

*As a nurse … you’re the one who has to deal with the skin sloughing off and the really awful emotional stuff and the parents crying beside the bed your whole shift, you know what I mean, you don’t get to get away from that. (Neonatal nurse HCP 27).*

*We don’t enjoy doing any of the things we have to do to them (Junior neonatal nurse, HCP 12).*

A senior neonatal trainee expressed an equal amount of distress. Her perception that there are few good outcomes is not supported by the data, but may impact on counselling parents (here discussing 23 and 24 week gestation babies):

*Here we’ve got this awful situation that’s going to, in the best case scenario, condemn you to another 16 weeks of living with us all day every ….the issue of informed consent is tenuous at best….I think for me I think we do a lot of horrible things to very, very small people and lots of horrible things to families with very small risk of good outcomes in that situation (Senior neonatal trainee, HCP 9).*

### Uncertainty of outcome

Despite the negativity towards resuscitation of periviable babies that was seen throughout the data, many of the neonatal and paediatric staff had experience of babies who had done better than they had thought possible during their perinatal course:

*I’ve had kids come back that have surprised me… that I really thought were going to have severe impairment either at the time of birth or during their time here and they’ve really surprised me (Experienced neonatal nurse, HCP 12).*

A senior neonatologist described a patient where an unexpectedly good outcome has changed his certainty in prognosis for individual babies and his practise. This self-reflection was not stated by many HCPs:

*I don’t think we can always predict what’s going to happen. That’s probably changed my practise a bit, seeing that boy grow up- just because you see something bad on the scan it doesn’t necessarily mean that everything is going to be atrocious. (Neonatologist, HCP 10).*

One paediatrician who specialises in children with developmental problems discussed how most of the children that she sees at later follow up appointments are not severely affected with long term sequelae:

*They are seeing me because they have problems… common problems that I see with those kids are learning problems and a few of them would have cerebral palsy, severe ones, not that many. (Paediatrician, HCP 16).*

Good progress with intact development in extremely preterm babies led this regional paediatrician to feel uncomfortable offering palliation for 24 week gestation babies where they have to provide care awaiting retrieval, even where the teaching is that these babies are less likely to do well than babies born in the tertiary unit:

*If the parents say that ‘I don’t want you to resuscitate my 24 week baby’, I would feel very uncomfortable actually because I’ve seen them doing so well… If they are obviously born in good condition, you want to give them the best go. (Paediatrician, HCP 31).*

### Disability in remote places

Many of the patients cared for in the tertiary facility reside in regional or remote locations. Where potential disability could be a burden, this obstetrician was concerned about the distance from large health facilities:

*People from remote areas, you need to keep in mind what’s going to happen to the baby once it’s born…That will influence me that a morbid baby is not going to do very well, or be high needs in western Queensland. That family sometimes will need to move to a place close to a major centre and it can wreck their lives. (Tertiary obstetrician, HCP 25).*

Interestingly, in contrast, this paediatrician who works in remote locations suggests that a child with a disability may find more acceptance in a smaller remote centre despite disability because there is less negative judgement placed on disability:

*In the remote communities, a lot of families do accept children with delayed milestones and whatever, they are accepted and the expectations are not as much as city folk (Remote paediatrician, HCP 29).*

### Differing disciplinary perspectives

Differences in perception between the neonatal counsellor and the obstetric counsellor were evident in the data. An obstetrician stated that the neonatologist does not dwell adequately on the negatives of disability, and instead talks about potential positive outcomes:

(The neonatologists) *talk about the positives, not the true 24 h a day, 7 days a week morbidity they truly will be faced with if they have a damaged surviving baby (Tertiary obstetrician, HCP 25)*

A neonatologist had the view that active steps to optimise the potential for a 23 week gestation baby needed to occur before he would consider counselling the parents about whether to resuscitate or not, whilst the obstetrican would not administer steroids until the neonatal consultation had occurred:

*They requested me to go and see the mum who is 23 weeks. I say, are you going to give mum steroids? They said no. I said in that case I don’t need to go and see her…..it’s kind of a little bit almost inconsequential for us to be involved if the baby is going to be compromised even before birth. (Neonatologist, HCP 7).*

### Influence of personal experience

Throughout the data, staff added reflections from a personal perspective. Some staff had considered the possibility of pregnancy complications for themselves and how this might influence their opinions:

*Am I giving them objective enough information to help them to be able to make a decision without saying ‘yes I think we should do everything’, because my own fear is getting in the way? But also I think, you can’t help but think from my point of view if I was in that situation…it’s something that is very commonly discussed, particularly amongst O and G registrars because bad stuff always happens to us in pregnancy. (Obstetric trainee, HCP 30).*

An older neonatal nurse recognised that her views have changed with time:

*When I was young I would have said that my partner and I wouldn’t have ever managed with a disabled kid…since I’m older now I would have loved my child regardless of what they were like and I know that I would have handled whatever. (Neonatal nurse, HCP 27).*

The recognition that personal experience may change perspectives was displayed by this junior paediatrician. When initially interviewed, she felt that intensive care should not be provided for babies under 25 weeks gestation and she would not want her own baby to be resuscitated under 27 weeks. A year after initial interview she commented thus:

*Immediately upon becoming pregnant and ‘seeing’ the baby on an ultrasound, it was like a switch had been flicked. Whilst I had been so adamant on my views in the past regarding resuscitation as well as termination of neonates with congenital anomalies, I found myself having had a complete 180. I found myself counting down the days to 24 weeks and on the day of announcing to my colleagues that should the baby present herself early, I would expect them to engage in full resuscitation with whatever this required. (Paediatric trainee, HCP 4).*

Participants were asked about their personal experiences of disability. Few had siblings or close contact with disabled people. An obstetrician did have a sibling with moderate disabilities. He did not feel his experience influenced his counselling, although he continued:

*I can see the effect that it had on my family, I don’t let that impinge on my counselling I don’t think….it tends to be about making people – giving people true awareness of what it means. (Obstetrician, HCP 17).*

### Evolving implicit bias

Senior clinicians often had more dogmatic certainty about whether babies should be offered active care. More junior staff were less aware of the expected outcomes, although they may spend more time with the pregnant women and the babies. A junior midwife and obstetric registrar commented respectively:

*I don’t get to see the babies down the track. I can just go from what we are told really, because we don’t get to see the end part (Midwife, HCP 24).*

*I don’t have the knowledge to go into the finer details about what sort of long term disability or impairment an extremely preterm baby might have. I don’t know it or I don’t feel comfortable discussing it because I just don’t have the experience (Obstetric trainee, HCP 30).*

An experienced trainee neonatal doctor had been expected to provide counselling at a centre without senior support earlier in her career:

*Year one of training … and you need to go and talk to these parents who are about to deliver a 25 week. Of course that was incredibly confronting because what on earth do you say to people in that situation…I was a youngster at that point myself and I wasn’t sure what to do (Senior neonatal trainee, HCP 9).*

Junior doctors preferred unambiguous guidelines to avoid the requirement for any decision making at different gestations:

*It’s too much guilt and pressure to put on families. That’s why we should have a strict policy on ‘below this we don’t resuscitate. That’s our policy’. (Junior paediatric trainee, HCP 4).*

The disconnect between the survival and outcomes statistics learnt by more senior staff during their training, and current literature was recognised:

*There’s a lot of work being done to improve outcomes, so if you speak with the more senior obstetricians – when they started practise their survival was 28 weeks (Neonatologist, HCP 8).*

## Discussion

This grounded theory study identified that attitudes of health care professionals concerning extreme prematurity were influenced by discipline specific implicit bias towards extremely preterm babies due to the risk of prematurity related disability. For some HCPs in this study, disability is perceived as a burden which no parent should risk and disability can be prevented by allowing all at risk babies to die. Parents were deemed to be too emotionally involved to objectively assess the risks for the foetus in peril. Hope and positivity were perceived as negative factors which prevent the family from opting for palliation. Implicit bias was expressed by the language used, for example where the parents should be counselled on the ‘right thing to do’ and ‘proper counselling’, both of which were linked to the belief that parents should decline resuscitation below 25 weeks gestation. HCPs in both the antenatal and neonatal care domains expressed feelings of guilt for playing a part in the survival of disabled children. Rarely did a clinician explicitly state that disabled children have less value as people. However, participants frequently stated that the disabled child may exert an intolerable burden on the family, affect relationships and even cause families to need to leave their homes in the quest for medical care for the disabled child. Genuine compassion was noted in the desire to protect parents from emotional harm and the baby from suffering. Paediatricians particularly appeared most positive about the future function of many of these families regardless of the outcome for the child.

Thus, data from this analysis suggests that a subconscious bias exists, which is moulded by the background and experience of the clinician. Role specific differences were evident in the form of negative prognostic messaging. Negativity about the long-term mortality and morbidity of babies born at extremely preterm gestations is found repeatedly in other studies [19–21]. These studies also reflect that obstetricians are more negative than neonatologists in their knowledge of survival rates and morbidity, and obstetricians may be less inclined to suggest that the baby receives active care as a result [35]. Where there is disagreement between the obstetrician and neonatologist about whether a baby should receive active care, the outcome for the neonate is worse [35]. Greater accuracy in knowledge is found in units with a proactive approach to the perinatal management of more immature babies and this is associated with improved outcomes [36, 37].

Negative attitudes and moral values of HCPs influence decision making at periviable gestations [38], partly because of the inaccurate data given to the parents, and also in the message framing of the prognosis [39]. A review of cognitive bias and heuristics in medical decision making suggests that bias is under-investigated amongst medical personnel [40]. These studies, however, do not explore the origins of the negativity or reasons for the discrepancy between groups of clinicians. Our study confirms these differences in terms of role between those clinicians caring for the mother antenatally, the neonatal team and the paediatric team. In addition, experience changes the viewpoints of these groups of clinicians. Junior HCP are less certain and identify more closely with their patients. However, more certainty and paternalism in attitudes was seen in some of the more senior medical clinicians in this study.

The differences between the HCPs who provide care prior to delivery and those after the birth may be explained, in part, by data which emerged from this study. Obstetricians are expected to deliver good health care during pregnancy and ensure the delivery of a full term healthy baby. As shown in the data, exposure to terminations of pregnancy for abnormality occurs at even a junior midwifery level. The work of a junior midwife incorporates a role to support the patient, but they are expected to keep a distance emotionally from the patients distress at the termination of the affected foetus. For the senior obstetrician, a disabled baby because of prematurity, may be a personal failure. HCP with a primarily antenatal role were least likely to trust parents to make objective decisions. There were few variations in attitude between midwifery and obstetric medical groups.

The focus of HCPs involved in care of the baby is different to the HCPs caring for the mother prior to delivery. Some neonatal clinicians reflected that the intensive care required is so burdensome for the baby and the family, that palliation may be a preferable option. This may be a measure of the distress the clinicians themselves are experiencing, particularly where the clinician appears to feel guilty for helping a baby to survive who is later profoundly disabled. The nurses use of the word ‘disturbed’ when reflecting on a graduate of her care, may reflect her guilt at helping the baby survive, or perhaps that the disability itself is not deemed to be acceptable. This finding concurs with previously described moral distress arising from the care of sick small babies [41]. This unease is most noticeable in the staff with the closest day-to-day care of the neonate – the neonatal nurses, and the junior doctors. More senior neonatologists articulated the difficulty in prognostication for individual babies, and the need to be hopeful. It may be that this perspective provides justification for the suffering, whilst opining that ‘even the disabled have rights.’ Extremely premature babies will remain in the neonatal unit for months after delivery and the HCPs in the neonatal unit will form a relationship with the families based on shared care for the neonate [42]. Consistent with the literature, a difference in negativity was seen between neonatology medical staff and neonatal nurses [43, 44], and this may reflect a difference in the immediacy of day to day care, and social engagement with families. Families often visit the neonatal unit for many years following admission, and engagement with the family via social media has also enabled the staff to see babies progress. This does not often occur for many of the midwifery and obstetric staff who reported that they rarely know the long-term outcome of extremely premature babies. This may account for some of the differences seen between HCPs caring for families antenatally and postnatally and is an area which needs further research.

In contrast to our study however, Lavin et al. [45], in a large North American study, however, were able to show that obstetric and neonatal doctors were relatively consistent in their attitudes towards resuscitation except at the gestations below 23 weeks, and also relatively accurate in their knowledge of outcomes. Accuracy in knowledge and optimism towards resuscitation was also reported by Janvier et al. [36] with no difference between obstetric and neonatal trainees in this regard. Accuracy and positivity appear to lead to consistency to an active approach to management towards those babies of a lower gestation in the services studied than in Australian groups [8, 10]. Our study suggests that local variations in positivity seen between HCP roles may be a marker that the care of vulnerable pregnancies may be less proactive and potentially contribute to poorer outcomes amongst the most disadvantaged groups.

Conversely, paediatricians appeared most positive in their attitude towards extreme prematurity. The inclusion in our study of paediatric staff adds evidence that positivity about families and their future coping is warranted. Whilst most had minimal exposure to the extremely preterm baby at the time of birth, they offer care to these children until late adolescence. These paediatric opinions have rarely been included in the literature about attitudes towards extreme prematurity, and this study adds valuable information about their insights. The junior paediatricians appeared less likely to consider active care for extremely preterm babies to be appropriate than their senior colleagues. Trainees had a perception that most of these babies have disability whilst the more senior paediatricians reflected that they did not see many severely disabled children from prematurity. Paediatricians from regional and remote centres were positive about resuscitating smaller babies as they perceive that many will ultimately do well. The paediatric attitude is the most informed in terms of exposure to disability of the child and the effect on the family, and their relative positivity suggests that the more negative perinatal staff may need to consider that their outlook may not reflect the true consequences as seen by those caring for the children later.

Remote residence is often linked with the poor provision of health care resources [31, 46]. Some HCPs consider that active care should be considered at a higher threshold for these families. Conversely, exposure to working in remote areas was seen to modify one clinicians’ opinion, who confirmed that children with disability may be more accepted within the communities in these areas. Another clinician who has worked in remote areas confirmed that limited access to disability resources did not lead most families to leave the area. Perhaps acceptance into the community is more important to some families than having more medical resources [47]. Our study confirms that families remain in their communities and find ways to access the care their children need. Thus, families themselves should be involved in decisions where aspects of their life circumstances are considered germane to care offered.

Personal experience of disability was uncommon amongst the participants. However, some participants recognised that they had markedly changed their views as they have become older. One HCPs’ views changed markedly after her initial interview once she herself became pregnant. Some staff appeared to have reflected on how they personally would cope with a disabled child, and these participants seemed more accepting of disability. Empathy and acceptance seem to have occurred where self-reflection was found. This suggests that HCPs may benefit from these strategies being encouraged within their workplace.

Implicit bias in periviable counselling by neonatologists has previously been demonstrated by Shapiro et al., who suggest that clinicians who show negative bias towards socioeconomic status were more likely than those who did not show bias to recommend palliative (comfort) care when presented with a patient of greater socioeconomic status [48]. The authors hypothesised that this could be because the clinicians identified more closely with these patients and that this reflected what they would choose for themselves. Our study suggests that this finding may be rather a result of implicit bias against the risk of disability, which those clinicians would consider unacceptable.

Previous studies have linked implicit bias to racial minorities [27, 28], obesity [49] and gender [50], all with negative implications for patient outcomes. Implicit bias towards the risk of disability, as is seen in this study, should be added to this list and needs further exploration in terms of patient outcomes. It is incumbent on HCPs to identify and be aware of their biases and may need specific training in order manage these [29]. Parents ask for hope and honesty from their HCP [51, 52]. Negativity induced by the implicit bias towards the extremely preterm because of potential disability may remove hope and thus potentially do harm. The overwhelming majority of parents in this region will opt for full active intensive care for their babies (30)and implicit bias among HCPs may impede their enjoyment of the babies, in situations where they have received a negative view of the long term prognosis.

Identification of personal bias is important in counselling parents antenatally using a model of shared decision-making [53–55]. The suggested models presented by Sullivan, Lantos, Haward and others remind the practitioner to reflect on biases they may have towards race, socioeconomic status and disability prior to meeting with parents in need of antenatal counselling. Implicit bias towards extreme prematurity related to the role of the practitioner must also be sought.

### Strengths and limitations

A strength of the study is that it has included participants who represent a range of experiences and disciplines involved in the care of periviable babies. Most of the senior clinicians in the tertiary service engaged in the interviews, with good representation at all levels of role and experience. The inclusion of paediatricians added information to the study because of their role in being able to review the longer-term implications of extreme prematurity, and is an unusual inclusion in a study of this nature.

The study did not aim to investigate the differences in attitudes of various groups of HCPs, these differences emerged from the analysis of the data obtained when studying attitudes of HCP towards extreme prematurity. The constructivist methodology allowed exploration of this category as interviews progressed. This is both a strength of the methodology, but a limitation as deeper exploration may have been possible in a more focussed study.

A further strength of the study was the constitution of the research team. Apart from the PI, the team included a bioethicist who has studied ethics in the medical and paediatric fields, a senior university academic who specialises in qualitative research and previously worked in midwifery but who reflects regularly on the potential for bias in her work and a senior university academic with a background in general practice who has published extensively in qualitative research and reflects on potential bias. The team provided a dispassionate group of opinions.

Limitations of the study include the geographical restriction of the study to three centres in North Queensland. Some of the findings may be relevant only to the area under study. Transferability of the findings needs to be considered in the specific context of other localities.

A further potential limitation of this study is the role of the primary investigator as a neonatologist working in the tertiary unit. She herself has opinions about the provision of active care for periviable babies, has researched their outcomes and is more positive than most of the participants, although aware of her biases. In addition, she knows all the senior participants having worked with them for several years. As a consequence, in an attempt to mitigate bias, interviews with many clinicians were done by a third-party unknown to the participants. Coding and analysis was done by the research group in conjunction with reflexive memoing.

## Conclusion

Role dependent implicit bias can occur in some HCPs who care for families at risk of extremely preterm birth. Implicit bias may be a cause of inappropriate negativity in antenatal counselling and explain role-dependent differences in negativity as influenced by the function of the role itself. When implicit bias is present, the clinician will be more negative in their counselling and message framing. Identification of the positive benefits of resuscitation may not occur when an emphasis on disability is maintained. As a result, vulnerable families may not receive an accurate perception of the possible outcomes for their baby during antenatal counselling. Self-identification of implicit bias, and non-judgemental institutional efforts to enable staff to recognise their biases and correct these would help in shared decision-making with parents to ensure that appropriate decisions are made from the family’s perspective. It is important that all HCP are aware of the accurate data for outcomes for babies, and that the influences of their own biases have the potential to affect families in decisions and function on the neonatal unit. Further research is needed to investigate whether negativity in attitudes persist when clinicians become aware and address their bias against extreme prematurity, and whether this in turn improves outcomes for our smallest patients.

## Supplementary Information

**Additional file 1.** Updated question guide. This file contains the semi structured question guide used by the interviewer. A semi structured format was used, with conversational approach, and questions used in the order which seemed appropriate for the interview. Please note that the questions cover a larger project, and only one category of results is found in this manuscript.

## Data Availability

The data is stored on the university storage system. The datasets generated during the current study are not publically available as they are contained within interviews which enable identification of the participants. The whole data is also only a small part of the categories of results obtained and therefore mostly not relevant for this paper. Coded data showed the results as published in this paper.

## Abbreviations

HCP: Health care professionals
NICU: Neonatal intensive care unit
MFM: Maternal foetal medicine
