# Supplementary material for: Exploring implicit bias in the perceived consequences of prematurity amongst health care providers in North Queensland – a constructivist grounded theory study
Source: BMC Pregnancy Childbirth. 2021 Jan 13;21:55. doi: 10.1186/s12884-021-03539-5 (PMC7805144; doi:10.1186/s12884-021-03539-5)
Supplement: Supplementary file 1 — Additional file 1. Updated question guide. This file contains the semi structured question guide used by the interviewer. A semi structured format was used, with conversational approach, and questions used in the order which seemed appropriate for the interview. Please note that the questions cover a larger project, and only one category of results is found in this manuscript. [file 12884_2021_3539_MOESM1_ESM.docx]

Please note:

This outline of the semi structured interview questions was requested by the journal. The manuscript submitted includes only one category of results of the analysis of the data. As analysis followed Charmazs’ grounded theory principles, the categories do not pertain to any single question. Further publications are expected.

As a semi structured format was used, the interviewers were not restricted to the questions asked and the interviews evolved in a conversational manner, with the order of questions as appropriate to the individual interview.

Question guide for staff attitudes study

(note this is a guide for interviewer)

Review consent for study and consent for recording

Review counseling options

Establish experience and role of participant in the workplace

*(eg midwife for x years)*

What experience have you had in talking to parents who are expecting an extremely premature or vulnerable pregnancy?

*(both formal as part of counseling and also witnessing information sharing or talking to patient outside formal counseling session)*

What gestation do you consider is appropriate for the offer of intensive care to be made to parents by the neonatal service?

Why?

The risk of severe disability under 27 weeks is approximately 20%.

“The guidelines for managing premature babies in Queensland, suggests that we should not offer any intensive care to babies under 23 weeks gestation, that parents should have final say if we resuscitate the baby from 23 to 25 weeks, and that we should resuscitate over 25 weeks unless there are other factors eg congenital abnormalities”.

What do you think about the guidelines?

Who do you feel should make the final decision about whether to offer care to babies who will need intensive care after birth?

Why?

Are there any non-medical factors which you think should be taken into account in initially offering intensive care? Eg ‘the precious baby, or where all other kids in care

What do you think most parents know about prematurity before they face the prospects of extreme prematurity, and do you think they are able to give informed consent to allow their baby to be resuscitated?

One of the options we give to parents, is to see ‘how the baby is’ at birth, initiate intensive care, but then review how the baby is doing in the first few days with a view of withdrawal if not doing well. Have you seen this occurring? How does this seem as an approach?

*(question only relevant for workers in neonatal unit)*

What about babies with known anomalies eg Downs, hydrops? – is this different to extreme prematurity in terms of who should decide to proceed to intensive care – why?

What is you experience of disability outside of hospital?

Are you religious, and does this help shape your opinions?

Would you choose to have your baby resuscitated at 23/24/25 weeks gestation?

Is there anything which you would like to say about this topic?
